# A TRADITIONAL HEALING PROGRAM ON THE NAVAJO NATION DURING COVID

**DOI:** 10.1093/geroni/igae098.1469

**Published:** 2024-12-31

**Authors:** Karen Kopera-Frye, Karen John, Robynn Frank

**Affiliations:** New Mexico State University, Las Cruces, New Mexico, United States; New Mexico State University, Las Cruces, New Mexico, United States; New Mexico State University, Las Cruces, New Mexico, United States

## Abstract

Tribal Critical Race Theory (TribalCrit) posits three types of knowledge: 1) cultural knowledge or tribal ways including traditions and Indigenous Ways of Knowing (IWOK); 2) knowledge of survival involving how and why change and adaptation can be beneficial to the community; and 3) academic knowledge often referred to as ‘‘book smarts.’’ IWOK and academic knowledge are critical for survival and improving the lives in Native communities. This project can best be thought of as a variation of Community-based participatory Research, or Community-based participatory advocacy. Two Navajo students and a faculty member created a project which would use traditional healing community values to understand how COVID had disrupted cultural ways, including IWOK. A Navajo program, beginning in 2007 called “Restoring and Celebrating Family Wellness (RCFW)” involved an organized tribal summit to hear from community members about their concerns, issues, and priorities via workshop venues called “Open Space”. Beginning in 2008, monthly workshops were held including elder presentations, storytelling, and health concerns addressing community needs of violence prevention and raising balanced children. Due to COVID, the workshops ceased in 2020 when the reservation was locked down. Subsequently, with the vaccines available, the project involved reviving RCFW. The Navajo community had experienced social isolation due to an inability to gather and share daily life experiences. Deaths of elders resulted in the loss of wisdom keeping elders and devastated the community. This revived program was successful in improving well-being and increasing connectedness among the Native community, thus restoring IWOK and demonstrating community resilience.

